# Cancer Metabolism: The Role of Immune Cells Epigenetic Alteration in Tumorigenesis, Progression, and Metastasis of Glioma

**DOI:** 10.3389/fimmu.2022.831636

**Published:** 2022-03-22

**Authors:** Kouminin Kanwore, Konimpo Kanwore, Gabriel Komla Adzika, Ayanlaja Abdulrahman Abiola, Xiaoxiao Guo, Piniel Alphayo Kambey, Ying Xia, Dianshuai Gao

**Affiliations:** ^1^ Xuzhou Key Laboratory of Neurobiology, Department of Neurobiology, Xuzhou Medical University, Xuzhou, China; ^2^ Xuzhou Key Laboratory of Neurobiology, Department of Anatomy, Xuzhou Medical University, Xuzhou, China; ^3^ Faculty Mixed of Medicine and Pharmacy, Lomé-Togo, University of Lomé, Lomé, Togo; ^4^ Department of Physiology, Xuzhou Medical University, Xuzhou, China

**Keywords:** glioma, glioma stem cell, metabolism, immune response, metabolism

## Abstract

Glioma is a type of brain and spinal cord tumor that begins in glial cells that support the nervous system neurons functions. Age, radiation exposure, and family background of glioma constitute are risk factors of glioma initiation. Gliomas are categorized on a scale of four grades according to their growth rate. Grades one and two grow slowly, while grades three and four grow faster. Glioblastoma is a grade four gliomas and the deadliest due to its aggressive nature (accelerated proliferation, invasion, and migration). As such, multiple therapeutic approaches are required to improve treatment outcomes. Recently, studies have implicated the significant roles of immune cells in tumorigenesis and the progression of glioma. The energy demands of gliomas alter their microenvironment quality, thereby inducing heterogeneity and plasticity change of stromal and immune cells *via* the *PI3K/AKT/mTOR* pathway, which ultimately results in epigenetic modifications that facilitates tumor growth. PI3K is utilized by many intracellular signaling pathways ensuring the proper functioning of the cell. The activation of *PI3K/AKT/mTOR* regulates the plasma membrane activities, contributing to the phosphorylation reaction necessary for transcription factors activities and oncogenes hyperactivation. The pleiotropic nature of *PI3K/AKT/mTOR* makes its activity unpredictable during altered cellular functions. Modification of cancer cell microenvironment affects many cell types, including immune cells that are the frontline cells involved in inflammatory cascades caused by cancer cells *via* high cytokines synthesis. Typically, the evasion of immunosurveillance by gliomas and their resistance to treatment has been attributed to epigenetic reprogramming of immune cells in the tumor microenvironment, which results from cancer metabolism. Hence, it is speculative that impeding cancer metabolism and/or circumventing the epigenetic alteration of immune cell functions in the tumor microenvironment might enhance treatment outcomes. Herein, from an oncological and immunological perspective, this review discusses the underlying pathomechanism of cell-cell interactions enhancing glioma initiation and metabolism activation and tumor microenvironment changes that affect epigenetic modifications in immune cells. Finally, prospects for therapeutic intervention were highlighted.

## Introduction

Gliomas are large brain and spinal cord tumors associated with three types of glial cells (1, 2), astrocytoma (astrocyte, found in cerebrum and cerebelum), ependymomas (ependymal cells found in ventricules and spinal cord), and oligodendrogliomas (oligodendrocytes found in cerebrum). In some cases, these gliomas can combine and affect several glial cells (example, aligodendrocytomas-astrocytomas) (3, 4). Gliomas differ from other brain cancers by their origin (glial cell), aggressiveness with high mortality (15 month maximum), resistance to drugs, and grade scales (one to four) (5). Recent findings have demonstrated that gliomas, especially glioblastomas formations, are initiated by the alteration in the genome and epigenetic modifications of glial cells due to age, radiation, and microenvironment (6, 7). The altered genome and microenvironment trigger glial cells’ excessive proliferation and increased glycolysis *via* the *PI3K/AKT/mTOR* pathway and the Warburg effect. These promote epigenetic modification mediated by histone acetylation and DNA methylation, which are characteristics of glioblastoma multiform (the highest grade glioma) (8, 9). Also, upon injury, hypersecretion of proinflammatory cytokines such as *interleukins (IL-6)* and *tumor necrosis factor (TNF)* have been shown to contribute to carcinogenesis and impede the destruction of cancerous cells (10, 11). Furthermore, while *transforming growth factor-beta (TGF-β)* family has been reported to play crucial roles in the embryogenesis and differentiation of the nervous system (12, 13). However, recent works have found *TGF-β* overexpressed in gliomas, where they are implicated in facilitating the proliferation and regeneration of cancer cells (14–16). Intriguingly, Zhang et al. and others have also demonstrated the essential roles of *TGF-β* in the polarization of macrophages into tumor-associated macrophages (17, 18). In particular, works from Keshamouni VG’s reported that members of *TGF-β* family proteins bind to macrophages to induce their reprogramming through the polarization change and alteration of plasma membrane receptors and peptides necessary for the recognition of cancer cells, thereby repressing anti-tumor responses from macrophages (19–21). As such, by promoting the evasion of cancer cells to immune surveillance, cancer cells proliferations are hastened when the aforementioned proinflammatory responses are hyperactive. With evidence that the hypersecretion of *IL-6, TNF*, and *TGF-β* scaffolds cascades that promote evasion of immunosurveillance by glioma cells, it is speculated that the development of new therapeutic approaches that combine radiotherapy/chemotherapy and immunotherapy might circumvent immunosurveillance evasion and help target glioma progression effectively.

From an oncological and immunological standpoint, this review briefly discusses cell-cell interactions enhancing gliomas initiation and metabolism activation and tumor microenvironment changes that affect epigenetic modifications in immune cells. Also, perspectives and recent progress in the exploration of therapeutic intervention were highlighted.

### Niche and Energy Demands of Gliomas in Brief

The glioma stem cell niche significantly contributes to tumor cell proliferation and propagation and promotes tumor initiation due to their ability to self-renew, regeneration of the transient population of growth cells, and resistance to therapies.

Reportedly, four stem cell niches and microenvironment, namely, perivascular, hypoxic, invasive, and acidic niches, contribute to tumor maintenance and progression. These niches promote the genes that regulate cell stemness. Specifically, hypoxic and perivascular niches have been associated with upregulated expressions of *hypoxia-induced factor 1 alpha (HIF-1α)* and *vascular endothelial growth factor (VEGF)* (22). It has been reported that an acidic niche promotes *VEGF* gene upregulation which tends to support tumor vascularization and growths that promote malignant glioma cell metastasis along neurons propagation directions (23) and tumor progression (24). Xie Q et al. demonstrated that the invasive niche promotes glioma stem cell invasion and reduces their proliferation ability according to the “go or grow” hypothesis (25). Also, they demonstrated that a reduction in glioma cell proliferation significantly improves the glioma cell invasion (25). In consolidation, Hoang-Minh LB et al. also demonstrated that metabolic genes regulate tumor recurrence after clinical resection and that slower glioma-proliferating cells are underlying factors for tumor heterogeneity, progression, and recurrence (26). The ability of these glioma cancer stem cells to regulate progenitor cells in neoplastic tissue contributes to tumor bulk and development. Most tumors generate their energy by glucose fermentation and glycolysis (anaerobic and aerobic glycolysis) in the mitochondria *via* oxidative phosphorylation reactions to drive tumorigenesis (Warburg effect) (27, 28). Cancer cells prefer this type of metabolism due to the high demand for energy for their rapid proliferation and adaptive capacity to colonize diverse microenvironments. Notably, there are intrinsic heterogeneities in cancer cells (metabolism and gene expression) as well as in the tumor (metabolism, cancer cell types, and plasticity). Also, extrinsic heterogeneity such as abundance metabolic substrates, different types of stromal (fibroblast and astrocytes), and immune cells influences or affect cancerous cells/tumor growth (29, 30).

### Incomplete Cell Division: *GDNF* Interaction With *SOX1*, *SOX2*, and Cyclins in Glioma Stem Cell Initiation

Cell division is a crucial and delicate process because any aberrations at any stage might have detrimental effects on the daughter cells (31, 32). As such, three checkpoints (G1, G2, and M phases) are put in place to circumvent any defects in the genetic makeup of daughter cells. At the G1 phases, where cyclins D ensures that cells progressing to the S phase have the essential requirement (33), *GDNF* has been found to play vital roles in stimulating and regulating the cell to enhance their transition to the S phase (34, 35). Also, during G2, where cyclins and *p53* crosscheck the perfection of DNA replication and the absence of any damage before the cell enters the M phase, the involvement of SOX family members (*SOX1*, *SOX2*), and *signal transduction activator of transcription 3 (STAT3)*, *CREB/CBP* has been shown (36, 37). Occasionally, cell division processes in anaphase are incomplete due to underlying idiopathic; however, these cells become dangerous because some retain the ability to multiply (38, 39). Since the cascades in anaphase ensure that the chromosomes are separated and migrated *via* the mitotic spindle, its incompletion results in unbalanced distribution of genetic codes in the cells. Ultimately, this causes gene mutation in daughter cells and increases the expression of the genes that regulate the cell cycle (40). The overexpression and interaction of cyclins and *cyclin-dependent kinases (Cdks)* regulate cell cycle progression by promoting the activation of proteins that enable the process to pass designated checkpoints (41). Reportedly, during the cell cycle, the levels of cyclin-dependent kinases remain stable while cyclin levels do change to promote the progression of the cell from one stage to another.

Cells that fail to undergo all the required processing in the anaphase cannot pass the M phase to complete their division (42). Consequently, during the subsequent cell division, G1 and G2 cyclins are overexpressed along with oncogenes that cause irreversible damage such as gene mutation, chromosomes crossing over, and breaking within the cells (33). In this instance, it has been shown that overexpression of growth factors such as *GDNF* and *SOX1* protects the abnormal cells against apoptosis, and the failure of P53 to repair the damage increases their risk of becoming a cancer-initiating cell and cancer stem cell (38). Typically, P53 will halt and repair damages occurring at the G1 phase; as such, it cannot cater to damages occurring at the anaphase and metaphase. Also, oncogenes are able to impede *P53* from triggering apoptosis of abnormal cells (38).

**Figure 1:**
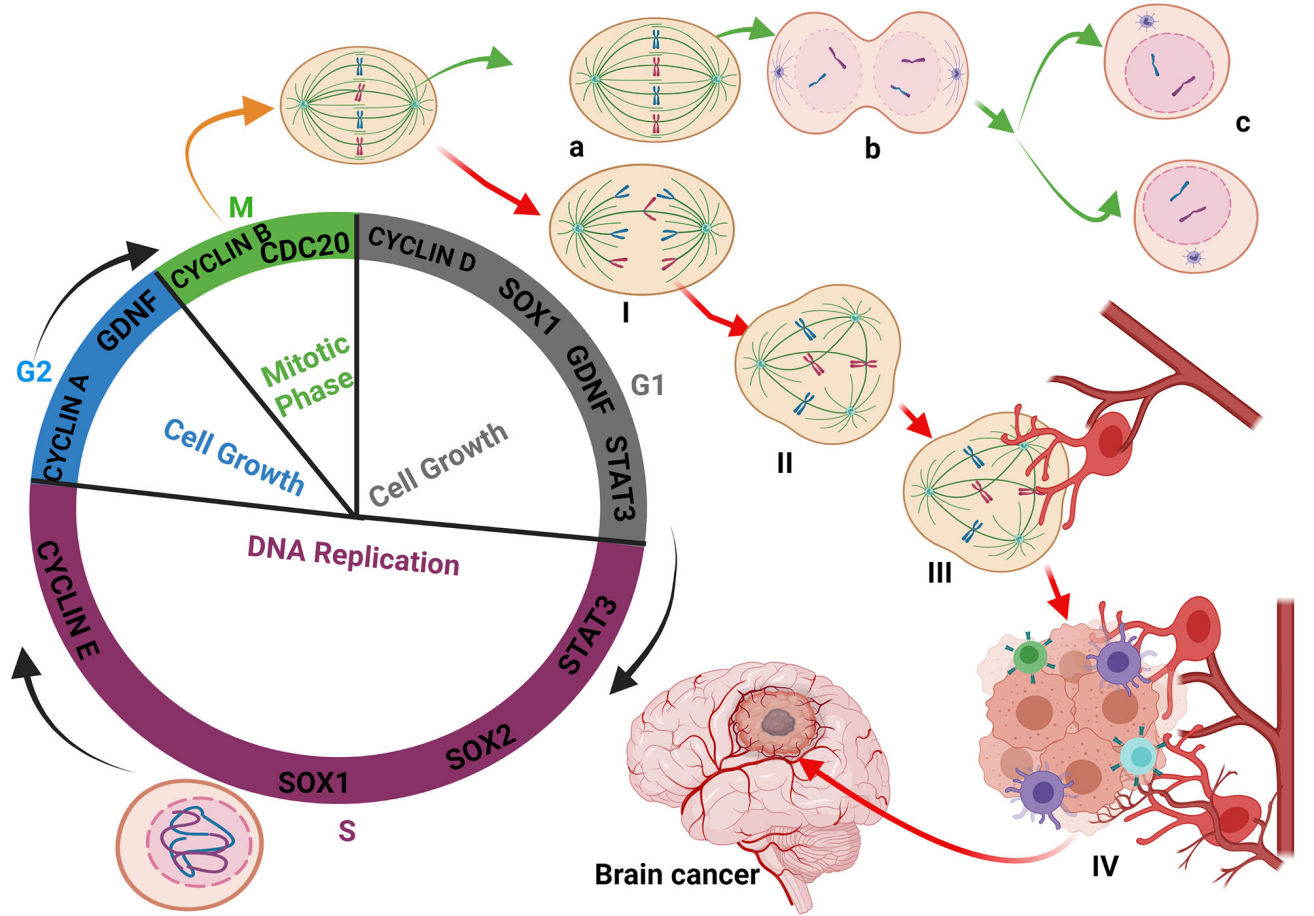
Glioma initiation: Interaction between the abnormal undivided cell and immune cells. During the cell cycle, the defective cell is not allowed to achieve division. Immune cells-like macrophages and T-cells destroyed the defective cell to prevent cancer initiation. Most of the oncogenes expressed during the cell cycle process are mainly Cyclins, GDNF, STAT3, and SOX family members to maintain the checkpoints and respect the timeline of cell division and integrity **(A–C)**. The fact that certain defective cells are blocked and cannot achieve their division stagnates and is continuously exposed and affected by the cell cycle oncogenes (I, II). The exposure of certain immune cells to GDNF reprogrammed and modified their polarity, leading to their activation and massive release of cytokines (III). The reprogramming of macrophages by the bonding of TGF-β family members on their membrane receptor retains the macrophages on their activated form. Sometimes, due to unknown reasons, the defective cell can migrate closer to the blood vessels and benefit from reprogrammed immune cells, which stimulate their multiplication (IV) through cytokine synthesis, activation, and modification of the cell’s metabolism leading to epigenetics modification and glioma initiation.

Besides gliomas initiations due to the alteration of genetic materials (epigenetics and mutation), other intra- and extra-cellular factors are involved. These include the overexpression of oncogenes during healing, tissue regeneration, nervous system differentiation during embryogenesis, and the development of organs (43, 44). *GDNF* plays a crucial role in facilitating the aforementioned growth and reparative processes. However, the fluctuation of its expression during inappropriate periods such as immunodeficiency, physiological stress, or during the recovery period preceding a severe illness encourages cell-cell interactions and prompt immune cell activation. During this period, an interaction between the abnormal undivided cell and an activated macrophage stimulates its growth and can lead to cancer initiation (**Figure 1**). The proliferation of cancer cells and their binding are facilitated by increased fibrin levels, which were meant for supporting immune cell activity. Therefore, the higher expression of fibrin contributes to cancer stem cells colony formations and the initiation of angiogenesis necessary for tumor formation, nutrient supplies, and metastasis. The growth of the tumor mass and its associated cells causes intussusception of the blood vessels, making its membrane fragile and allowing cancer cells to cross into the blood circulation and metastasize within the same or to the different organ(s) (**Supplementary Figures 1A, B**).

**Figure 2:**
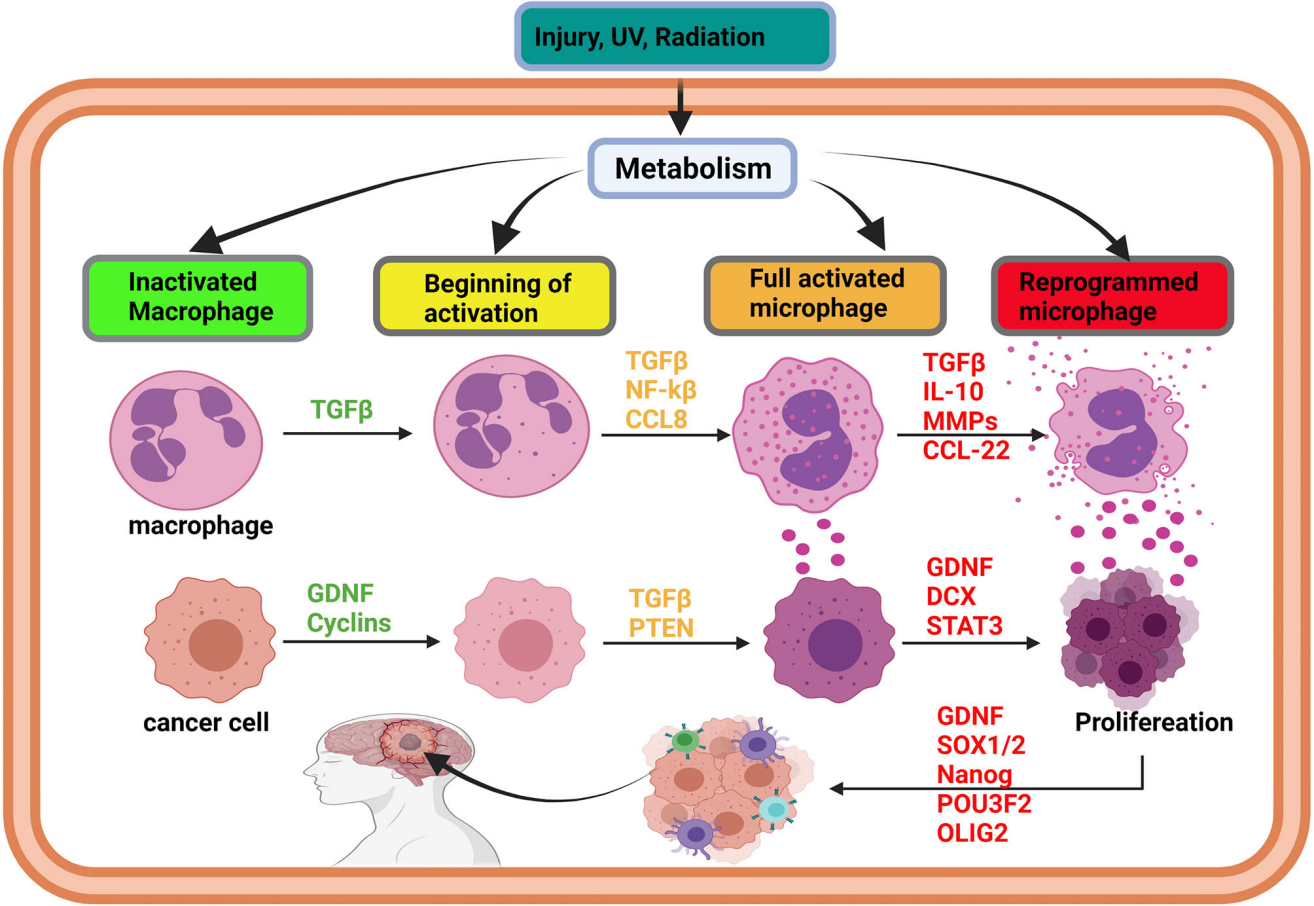
Initiation of glioma via immune cell reprogramming of cancer-associated macrophages. In brain injury, the activation of metabolism can affect the cells’ DNA and promote gene methylation and acetylation. The effects of metabolism on oncogene lead to methylation, acetylation, resulting in their overexpression. Simultaneously, overexpression of oncogenes, such as TGF-beta and IL-10, promotes macrophages’ activation and reprogramming, leading to the overexpression of cytokines by an immune cell. As long as the injury persists, TGF-beta family members and cytokine will be overexpressed to heal the injury. The synergistic effect of TGF-beta and cytokines can induce the transformation of the surrounding cell into a cancer cell and trigger proliferation. Cancer initiating cells can also be reprogrammed into stem-like tumor-propagating cells, spread in the brain, and cause glioma. Glioma initiation is mediated by intrinsic (metabolism, epigenetic modification) and extrinsic factors like U.V. and radiation.

SOX2 is one of the stem cell markers originating from the *sex-determining region Y (SRY)* superfamily group and *SOXB1* subgroup members with *SOX1* and *SOX3* (45). *SOX2* plays a critical role in neuronal cell formation and holds embryonic stem cells undifferentiated (46). It has been stated that the overexpression of *SOX2*, *Oct4, Klf4*, and *c-Myc* transform differentiated cells into induced pluripotent stem cell and their renewal by activating cell cycle processes (47). The cell cycle is vital for the renewal and proliferation of stem cells (48). In fact, Albright et al. and Zhang et al. have reported that *SOX2* gene inhibition promotes neurodegenerative diseases (49, 50). However, when properly expressed, *SOX2* actively induces and regulates the cell cycle through its interaction with *phosphorylated cyclin-dependent kinases (Cdks)* and *cyclins (A, B, D, and E)* (51). The *SOX2/Cdks/cyclin* complex can also cause self-renewal and proliferation of stem cells (51). *SOX2, SOX1*, and *SOX3* form part of *SOXB1* subgroups and have a similar and redundant genetic sequence (52). The redundancy between members of the *SOXB1* subgroup enhances their interactions with other genes involved in the renewal and proliferation of glioma stem cells (*SOX1, SOX2, and SOX3*)/*Cdks*/*cyclins*. Hence, the overexpression of *SOXB1* subgroup members in any tumor-initiating cell (differentiated cell) can turn the cell into a reprogrammed induced pluripotent glioma stem cell (*IPGSC*) that can differentiate easily into any type of brain cell and cause heterogeneity within a tumor. The possible interaction between *TGF-β* (*GDNF*) and *SOXB1* (*SOX1, SOX2, SOX3*) family members might explain the difficulties in regulating glioma stem cell recurrence after surgical tumor resection and drug resistance (52, 53). The speculation is based on the fact that *SOXB1* subgroup member can reprogram any tumor cell into a tumor stem or progenitor cell (**Figure 2**). Additionally, *GDNF* can also compliment this by facilitate their reprogramming into neural stem cells which can differentiate into several types of neurons in the brain and spinal cord. The differentiation of neural stem cells into brain or spinal cord neurons represent a risk factor in the central nervous system cancer progression resulting in cancer cell interaction with the other cells of the central nervous system. The change of neural stem cells micrienvironment modifies its metabolism to facilitate its differentiation (54–56).

Most research findings report on the role of oncogenes in cancer cell proliferation by enhancing the cancer cell cycle. In some instances, cancerous cells may not be ready to divide, but due to the overexpression of oncogenes, the cells progress in dividing without the necessary preliminary process, thereby resulting in chromosomes and DNA damages. As such, checking the cell cycle *via* flow cytometry using nucleus staining with propidium iodide (PI) or Dapi to count ploidy (aneuploid, diploid) mighty not enough. It is suggested that the repartition and form of those chromosomes be checked to ensure DNA integrity and predict the possible modifications in the next cell cycle. This will help to mimic tumor growth.

### Symbiotic Interaction Between Abnormal Incomplete Divided Cells With Immune Cells: Maladaptations of *GDNF*

Recent reports have implicated immune cells in playing vital roles during glioma initiations (57, 58). Also, it has been demonstrated that the abnormal symbiosis between cancer and immune cells increases the transformation of both cells (59, 60). For example, during tumor initiation, *GDNF* (a *TGF-β* family member) was found to be impeding anti-tumor immune cells activities but instead transforming the cells into tumor-associated immune cells (61, 62). *T-cell receptor-mediated immune cell (TCR)* nitrosylation inhibition and transformation are achieved by decreasing glycolysis and caspase activity, which regulates immune cell death (63, 64). While caspase activities are decreased, increases in *GDNF* levels tend to promote abnormal T-cell and tumor-initiating cell survival (65). Meanwhile, the nitrosylation of T-cell receptors, while *GDNF* is overexpressed, encourages their bindings to *epidermal growth factor receptor (EGFR)* on T-cell to promote immuno-oncology phenomena, which regulates the proliferation, polarization into M1 or M2 microglia and cytokine (*IL-4, IL-10, IL-13*) secretions (66). Furthermore, reports have indicated that the synergy of *GDNF* overexpression and hypersecretion of interleukins trigger the polarization of tumor-associated macrophages into M2 microglia (67). Intriguingly, Zhang et al. had previously demonstrated that M2 microglial cells are found in the tumor microenvironment and were implicated in the participation of cascades facilitating tumor initiation, angiogenesis, and metastasis (68). The interaction between M2 microglia macrophages and abnormal incomplete divided cells highly increases the risk of glioma initiation due to alterations in the brain cell microenvironment resulting from cytokine overexpression. Influenced by the cytokine-altered microenvironment, glioma initiating cells begin to proliferation by raising their metabolism (glycolysis), thereby exacerbating inflammatory responses (69). Tumor-associated macrophages (M2) are capable of reprogramming fibroblasts into cancer-associated fibroblasts (*CAFs*) by increasing *IL-1β, EGF*, and *VEGF* (70–72). Interestingly, the symbiosis between M2 macrophages and tumor-initiating cells is essential for both survival and proliferation (**Figure 2** and **Supplementary Figure 2**).

### Change Metabolism Induces Epigenetic Modifications in Immune Cell and Scaffold Cancer-Initiating Cells and Metastasis

Metabolism is one of the essential phenomena contributing to human growth, reproduction, and life in general (73, 74). It basically involves the transformation of the specific substrate required for the activation of several genes and enzymes, which turn nutrients into simple molecules that the body can use. The involved mechanisms correlate cell metabolism, gene expression, and epigenetics modification (75, 76). Hence, cell metabolism and gene expression in healthy/unhealthy cells (such as immune and glioma cells) share many similar signaling pathways, and a minute change in their cell metabolism disproportionally influence several gene expressions (77–79). Among the signaling pathways, *PI3K/AKT/mTOR/GSK-3β* is the classical metabolism pathway involved in cancer cell growth (80, 81). Depending on the type of metabolic substrate, metabolic signaling pathways can switch from one to another to improve glioma cells’ survival and proliferation abilities. Over recent decades, glucose has been shown to be the essential nutrient for the human brain as its deficiency results in irreversible damage to the brain neurons (82–84). Mitochondria are responsible for glucose conversion into energy (*ATP/GTP*) (85, 86). Besides signaling *via PI3K/AKT/GSK-3β* regulating neural cell development and protein phosphorylation (80, 81, 87), it has been shown to regulate mitochondrial activities, specifically, glycolysis (87–90). Also, *GSK-3β* regulates mitochondrial metabolic function (biogenesis, bioenergetics, and apoptosis), mitochondrial structure (permeability), and mitochondrial dynamic (motility) (87, 90). Interestingly, cancerous cells (gliomas in particular) require all the aforementioned cellular function and makes use of glycolysis to meet energy needs for survival (91–93). Like other cells, alteration in the metabolism of immune cells can drastically change their functions and characteristics (94). Particularly in macrophages, metabolism affects their gene expression through *DNA* methylation and acetylation (95, 96). Similarly, histones *H3K9* and *H3K4* acetylation and *DNA* methylation have been reported to be involved in *GDNF* and *SOX1* overexpression in gliomas (50, 97). Thus, metabolism seems to exert similar effects on gene expressions in macrophages and gliomas. The hypersecretion of cytokines such as interleukins (*IL-6*) and tumor necrosis factor-alpha (*TNF-α*) due to hyperglycolysis provides additional evidence that changes in immune cell metabolism affect their gene expression and alter their functions (98, 99).

*IL-6* and growth factors (*GDNF, SOX1, SOX2*) play a critical role during inflammation, damaged tissue repair, cell proliferation, invasion, migration (100, 101). These genes expressions are related to cell metabolism, making the immune cell metabolic pathways the best candidate for the methylation of oncogenes and their overexpression in glioma. Also, the binding of *GDNF* to immune cell receptors (*EGFR*) promotes the activation of *mTOR*, which contributes to the improvement in immune cell metabolism, and increases epigenetic modifications (102). In consolidation of their findings, Yan et al. also recently reported that *PI3K/AKT/mTOR* regulates *HIF-1α*, which gets involved in reprogramming immune cell metabolism (99).

### Change Metabolism Induces Epigenetic Modifications in Cancer Cells and Scaffold Cancer-Initiating Cells and Metastasis.

**Figure 3:**
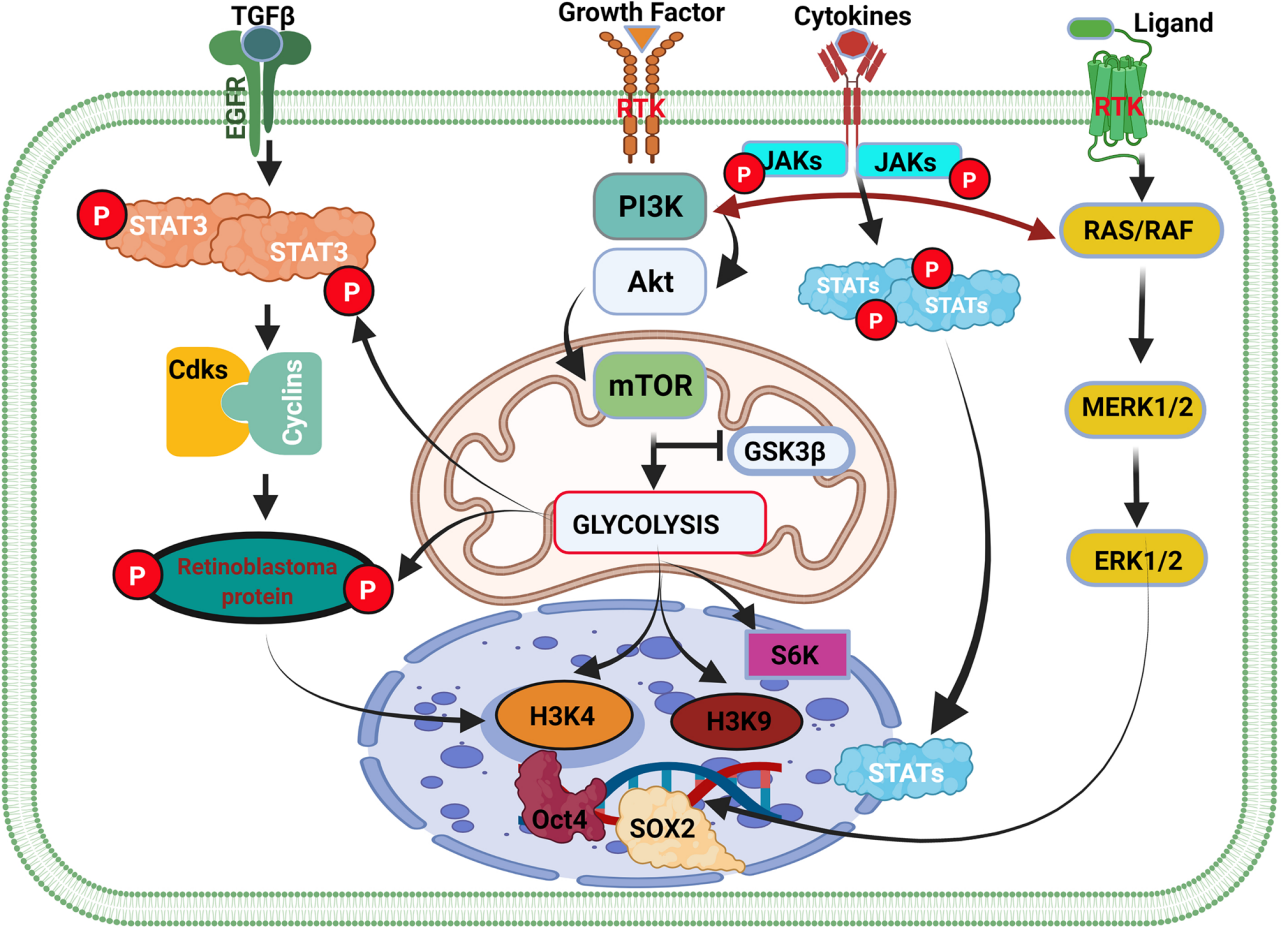
Cell cycle genes and the weaknesses associated with the initiation of glioma. Cell type transition and cancer cell initiation are a succession of events within the cell-mediated by external and internal factors. The overexpression of GDNF in normal cells contributes to the activation of the STAT3 gene and cyclin-dependent kinase (Cdks) binding with cyclin and their respective phosphorylation. The binding of Cdks-cyclin promotes the activation and phosphorylation of retinoblastoma protein (Rb) that stimulates the initiation of the cell cycle and cell division via DNA transcription. In inflammation, the extracellular overexpression of growth factors activates the PI3K/AKT/mTOR pathway, which controls glycolysis by antagonizing GSK3β activity. GSK3β activation promotes glycogen synthesis to promote the accumulation of glycogen that can be used later to produce energy for cell survival and division. GSK3β inhibition causes glycolysis to promote glioma initiation and epigenetic modification. In cancer, overexpression of GDNF or the activation of GDNF family ligands also promotes Ras/Raf signaling pathways leading to ERK1/2 activation and high expression. ERK1/2 translocate in the cell nucleus and regulate SOX1 family members’ overexpression. Activation of the metabolism pathway (PI3K/AKT/mTOR) regulates oncogene expression via gene methylation and chromatin acetylation (H3K4 and H3K9).

Like macrophages, *PI3K/AKT/mTOR* is the primary signaling pathway that controls the metabolism in glioma cells (**Figure 3** and **Supplementary Figure 3**) (99, 103). Dimitrova et al. and others have further shown that the *PI3K/AKT/mTOR* pathway controls glioma stem cell proliferation, invasion, angiogenesis, and metastases (104, 105). Additionally, they reported that *PI3K/AKT/mTOR/HIF-1α* plays a critical role in angiogenesis by inducing overexpression of *VEGF*. Indicating that *PI3K/AKT/mTOR* regulates/alters many functions in glioma cells and immune cells by controlling their metabolism (105). It is speculated that the difficulty in eradicating glioma might be due to the complexity of the *PI3K/AKT* pathway and the multitudes of genes reliant on this pathway. Many genes [including *EGF* and insulin-like growth factor 1 (*IGF-1*)] and hormones (including insulin) regulating essential physiological function rely on signaling *via PI3K/AKT* pathways.

Additionally, while *PTEN* has been said to antagonize *PI3K/AKT* signaling (106, 107), reports on the effect of *GSK-3β* on the pathway remain inconclusive. Yadav et al. and others stated that *GSK-3β* encourages the activation of the *PI3K/AKT/mTOR* pathway (108, 109). Nonetheless, Spokoini et al. had earlier reported that *GSK-3β* is antagonistic to the *PI3K/AKT* pathway (110). Even so, *GSK-3β* is known for its cell survival function, which aligns with promoting neuron survival and proliferation by PI3K/AKT signaling (111–113). Recently, Vashishtha et al. demonstrated that GSK-3 isoforms (*GSK-3α and GSK-3β*) were involved in the maintenance and progression of gliomas (114). They further showed that *GSK-3* was involved in the survival of glioma cells (114), which apparently contradicted what had been reported earlier (115). Thus, it is speculated that the specific role of *GSK-3* might depend on the isoform being expressed and/or their level of expression, as well as the metabolic pathways that might be involved in their genetic activation. Also, the fact that *PI3K/AKT* can switch among pathways that regulate metabolism and interaction with other proteins in immunes to alter their functions maladaptively just like in glioma, suggests that the pathway might be mediating other multiple underlying cascades leading epigenetic modifications (acetylation and methylation of histone) of immune cells and hastening the initiation and progression of gliomas.

As metabolism is an automatic process, its inhibition can lead to glial cell and neurons death. PI3K/Akt/mTOR inhibition to target cancer cell metabolism through drugs (such as Gleevec) has a side effect of affecting metabolism in a systemic fashion. The reduction of cancer cell metabolism leads to their death and necrosis, which has been shown to contribute to tumor maintenance. Hence, changing tumor metabolism slows its growth speed without reversing tumorigenesis. Moreover, gene sequencing such as *the cancer genome atlas (TCGA), next-generation sequence (NGS), single*-cell sequencing other transcriptomics only expose gene mutation, expression level, and identification of cancer cell hierarchy highly contribute to cancer therapy theoretically. On the other hand, even after detecting the exact mutations or alterations (amino acid sequences, nucleic base change, epigenetics modifications) that contribute to tumorigenesis and progression, it is difficult to induce any reversion due to the involvement of several factors such as the cell native environment which cannot be exactly mimicked under *in vitro* conditions. Additionally, the isolation process and tumor dissociation can cause stress to tumor cells alter their behavior. It is also important to acknowledge that cancer cells have multiple complex metabolism preferences. This metabolism heterogeneity makes cancer metabolism difficult to target because it can switch at any time using PI3K/AKT/mTOR or another unknown pathway.

### Warburg Effect Regulates Immune Cell Reprogramming to Facilitate Cancer Cell Metastasis *Via* Angiogenesis

Warburg effect or aerobic glycolysis is one of the metabolic mechanisms cancer cells use to accumulate energy (*ATP*) and proliferate for survival. Kornberg extensively reviewed the role of the Warburg effect in immune cell metabolic reprogramming (116). By the Warburg effect, oxidation of pyruvate to lactate (oxidative glycolysis) is preferred to oxidative phosphorylation for energy production even in the presence of oxygen. This form of modified cellular metabolism is typical of cancerous cells. The ability of the immune cells to adopt this altered mode of metabolism affects their cell lineage, which modifies immune responses and functions due to the impact of metabolism on regulating gene expressions. During inflammation, the role of immune cell metabolism determines the function of the immune cells in the progression or resolution of inflammation and disorder in autoimmune diseases. Kornberg et al. demonstrated that the anti-inflammatory functions of myeloid and lymphoid cells are regulated by the extent of aerobic glycolysis (117). In that, excessive aerobic glycolysis dampened the activation of anti-inflammatory responses. Evidently, the Warburg effect was shown in a recent work where it prevented inflammatory resolution by reprogramming and sustaining the activation of proinflammatory cytokines secretions and encouraged cancer cells progression (118). Furthermore, the Warburg effect has been shown to promote the methylation of inflammatory genes and the amplification of genes responsible for modifying immune cells’ plasticity and destiny (119, 120).

T lymphocytes such as *CD4* and *CD8* activation are linked to glycolysis, which regulates cytokine transcription and granule formation (119). The increase of *TGF-beta* expression in cancer cells and microenvironment affects lymphocyte metabolism *via* activation of the Warburg effect controlled by glucose transporters (*GLUT1* and *GLUT4*) and pyruvate dehydrogenase. This leads to lymphocyte function inactivation without deactivating the cytokines, which exacerbates inflammation and promotes cancer cell survival and proliferation. The exposure of cancerous cells to cytokine promotes tolerance and activates machinery to modify oncogenes expression necessary for the inactivation of macrophages (121, 122). The activation of *PI3K/AKT/mTOR* under hypoxic conditions regulates the hyperexpression *HIF-1* that regulates oncogenes upregulation through hypermethylation and histone acetylation (*H3K4* and *H3K9*) (123).

In other immune cells (natural killer, B lymphocyte, and dendritic cell) Warburg effect contributes to the activation and increase in oxidative glycolysis with high lactate production. The increase in lactate production and phospholipid metabolism regulates the migration and angiogenetic functions of immune cells as well as cancer cell metastasis (116). As depicted in **Figure 1**, the association of immune cells with cancer cells stimulates cancer colony growth. Malinarich et al. reported that glycolysis and oxidative phosphorylation (*OXPHOS*) in fatty acid oxidation modulates immune cell functions to enable them to attack abnormal cells (124). However, metabolic dysregulation might prevent the immune cells from performing their designated function. Also, cancerous cells have been shown to utilize inflammatory responses to facilitate their metastasis along the nerve propagation path *via* ventriculoperitoneal or blood vessels (23, 125, 126). Previous reports suggested that metastasis rarely occurs in glioma (127). Contrarily, recent findings have shown that glioma stem cells can contribute to glioma metastasis within and out of the brain. The ability of glioma stem cells to divide and differentiate contributes to glioma cell spread in the body even if it is not directly through blood vessels. Evidently, Hao et al. and others demonstrated that glioma cells could metastasize depending on the genes expressions (128, 129) and organelles dysfunctioning (130).

**Figure 4:**
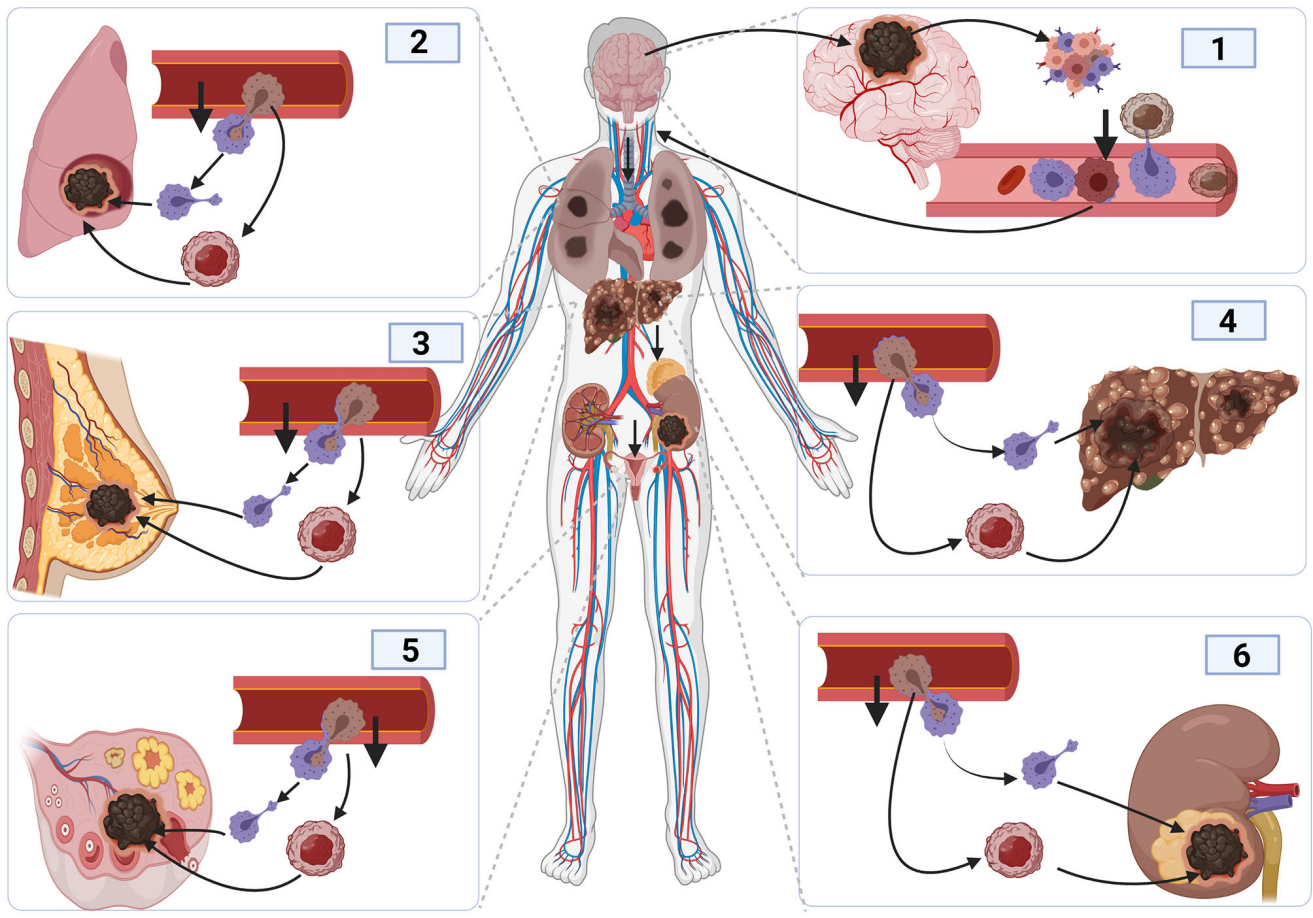
Cancer metastasis mechanism and tumor initiation and spread in other organs. The initiation of brain cancer promotes angiogenesis and tumor development, causing inflammation. The development of inflammation promotes immune response. Cancer cells bind to activated macrophages crossing the blood vessels, facilitating their migration into the blood vessel, and entering the blood circulatory system to reach other organs using the same process used to enter inside blood vessels.

Also, epithelial to mesenchymal transition (*EMT*) contributes to glioma cell metastasis (131, 132). The ability of glioma cells to switch from one cell type to another highly contributes to tumor invasion, leading to cancer cells metastasis (133). Cancerous cells associate with T-lymphocyte and dendritic cells to enable them to migrate in blood vessels (134). The activation of macrophages and dendritic cell during infection offers the cancerous cell the opportunity to move out of the vessel by employing the same mechanism as before (**Figure 4**). As such, cancerous cells tend to rely on the activation and inactivation of immune cells to facilitate their crossing in and out of blood vessels to enable them to metastasize (135, 136). Steinmetz et al. reported that radiotherapy could promote the transformation of glioblastoma cells into sarcomatoid metaplasia, which can cross the blood vessel and expedite the metastasize to other organs such as the liver (23, 137).

Furthermore, the appearance of new blood vessels facilitates glucose, minerals, and other nutrients supply that support cancer cell proliferation, tumor formation, a metastasis. The molecular mechanisms underlying angiogenesis are complex and still not completely elucidated; however, it has been shown to rely on the epithelial-to-mesenchymal transition (*EMT*) of immune cells and the polymerization of fibrin present in the tumor microenvironment (138).

### Cytoskeleton Polymerization and Epigenetic Modifications Scaffolded by Cell-Cancer Initiation

Hyperactivated metabolism initiated and regulated by the high proliferative effect of cancer cells, in turn, activates microtubule-associated proteins (*MAPs*). *MAPs* regulate the cytoskeleton polymerization *via* the modification of microtubule dynamics (139). The microtubule proteins (actins and tubulins) assembly scaffold a focal adhesion of cancer cells to their new environment to facilitate the transfer of metabolites and substrates necessary for cancer cells development. During glycolysis, the level of acetyl-coA increases with the high synthesis of citrate used for the Krebs cycle (140). Also, elevated citrate levels in the mitochondria induce their passive diffusion into the cytosol, where they are transformed into acetyl-coA that promotes epigenetic modifications (141, 142).

Further, acetyl-coA translocation into the nucleus where acetyl binds to histone (acetylation) to enhance target genes expressions. Additionally, the gene alteration caused by high acetyl-coA limits the activities of *P53* and cyclin upregulation, thereby impairing *DNA* damage repair and cell cycle regulation, respectively (143, 144). The alteration of oncogene leads to high expression of oncogenes and microtubule-associated proteins to enhance the transport of nutrients, substrates, and proteins. The synergy of the aforementioned phenomenon, inhibiting autophagy due to the hyperactivation of growth factor and *PI3K/AKT/mTOR* signaling pathway, stimulates cancer cells’ proliferation and tumor initiations and progressions. Furthermore, the development of dendrites requires cytoskeleton polymerization to increase the adhesive characteristic that makes phagocytosis easier for them. In addition, the development of microtubule filaments helps dendritic cells to migrate to their intended destination and mediate specific cell-cell interaction during antigen presentation. However, similar to cancerous cells, the cytoskeleton polymerization of dendrites requires energy acquired by the Warburg effect due to reprogramming in the microenvironment of cancerous cells (145). Ultimately, this goes a long way to affect dendritic cell lineage and modifies immune response and function because altered metabolism induces gene expression changes (77–79).

### Role of Lipids Metabolism in Tumorigenesis

Phospholipids play a crucial role in endoplasmic reticulum activities and regulate the phosphorylation of several proteins necessary for elevated oncogenes expression (146). Proteins such as phosphatidylinositol (3,4,5)-trisphosphate (*PIP3*) are associated with the cell membrane. The increase in *GDNF* (growth factor) is associated with *PTEN* that regulates phosphatidylinositol 4,5-bisphosphate (*PIP2*) phosphorylation into *PIP3*. *PIP3* binds to the Golgi apparatus, endoplasmic reticulum, and the nucleus membrane to facilitate transcription factor translocation inside the nucleus (147, 148). The phospholipids can also activate *AKT* and *mTORC2* to regulate glioma cell survival and metabolism, leading to cytoskeletal changes *via* the regulation of Rho protein and *CDC42* (147). Notably, phospholipids have been shown to regulate autophagy, phagosome, and lysosome, contributing to lipid metabolism and trafficking inside the cell (149). Even so, the overuse of phospholipids leads to alteration of the plasma membrane, affecting its permeabilization and reinforcement of the nucleus membrane to protect cancer cells against apoptosis (150).

Similarly, the plasma membrane of brain cells facilitates the flow (inward and outward) of nutrients, hormones, ions that keep the brain neurons alive. Nonetheless, its alteration facilitates massive influxes of calcium which stimulates the excessive synthesis of dopamine and causes overexcitation of the brain neurons (151). Additionally, the increase in intraneural calcium levels contributes to the cancelation of metabolism by interconnecting molecules and oncogenes *via* electron transfer (152). During the lack of glucose, lipids are transformed into glucose, which contributes to glycolysis and the Warburg effect. Together, these synergic metabolic activities highly contribute to cancer initiation and maintenance.

Due to its metabolic heterogeneity, glioma stem cell plasticity is an example of multiple molecular subclasses that plays several functions in heterogeneous tumors and cell populations (153). The metabolic and molecular heterogeneity is the cause of cellular diversity, morphological modification, adaptation, and resistance to therapy, thereby resulting in tumor recurrence after surgical resection, chemotherapy, and radiotherapy. The proliferation of glioma cancer stem cells requires high energy levels. Hence, the glioma cells can switch to lipid metabolism increasing in fatty acid oxidation to maintain their stemness and ability to differentiate into any cell type (154).

Lipids metabolism also contributes to immune cell reprogramming and immune response (155). *TGF-β* regulates fatty acid upregulation *via* lipids metabolism to stimulate cytokine hyperexpression during immune responses (156). The binding of growth factors such as *TGF-β* to immune cell receptors induces the activation of *PI3K/AKT/mTOR* and *PI3K/AKT/NF-kβ* signaling pathway, which contributes to cholesterol synthesis and inflammation *via sterol regulatory element-binding protein* (*SREBP*) (157). The activation of the *PI3K/AKT* pathway promotes the activation of *SREBP* that regulates the switching between cholesterol synthesis and proinflammatory cytokines such as *interleukine* and *chemokine* (157). High cholesterol level regulates immune cell endoplasmic reticulum and Golgi apparatus activities, contributing to epigenetic modification in immune cell *DNA* (158). Meanwhile, cholesterol synthesis is a fundamental requirement for several hormones and cytokines (159, 160). The conversion of cholesterol into hormones or cytokines contributes to lipid metabolism. Lipid metabolism is essential for *tumor-associated macrophages (TAM)* that produce more cytokine and chemokine necessary for cancer cell survival (161, 162).

### Perspectives and Advancements in Cancer Therapeutic Interventions

Nanotechnology is a new therapy strategy being used in the treatment of many diseases. The use of nanocarriers to support immune responses has become imperative in cancer therapy (163). This is because barriers created by the tumor and the presence of cocktails of protein and chemicals in tumor microenvironments induce immune cell reprogramming, which undermines their ability to fight tumors and reduce cancer cells population (164).

Herein, it is speculated that three therapeutic strategies might help make headways toward the fight and elimination of cancers. In brief, the first therapeutic strategy will be to develop interventions to break the barriers and penetrate tumor machinery. Secondly, delivery drugs or genetically trained immune cells that target inhibition, dysregulation, and/or destruction of cancer cells or functions/processes required for their survival. These trained cells could also be programmed to terminate dysfunctional inflammatory cells supplementing tumors with oncoproteins or facilitating a proinflammatory cytokine-rich microenvironment. Lastly, nanocarrier could be employed in the delivery of therapeutic to specific tumor microenvironment with the aim of effecting the two aforementioned strategies to inhibit their growth and progression. Deployment of these strategies might circumvent the side effects of intravenous chemotherapy and/or radiotherapy and improve treatment outcomes.

**Figure 5:**
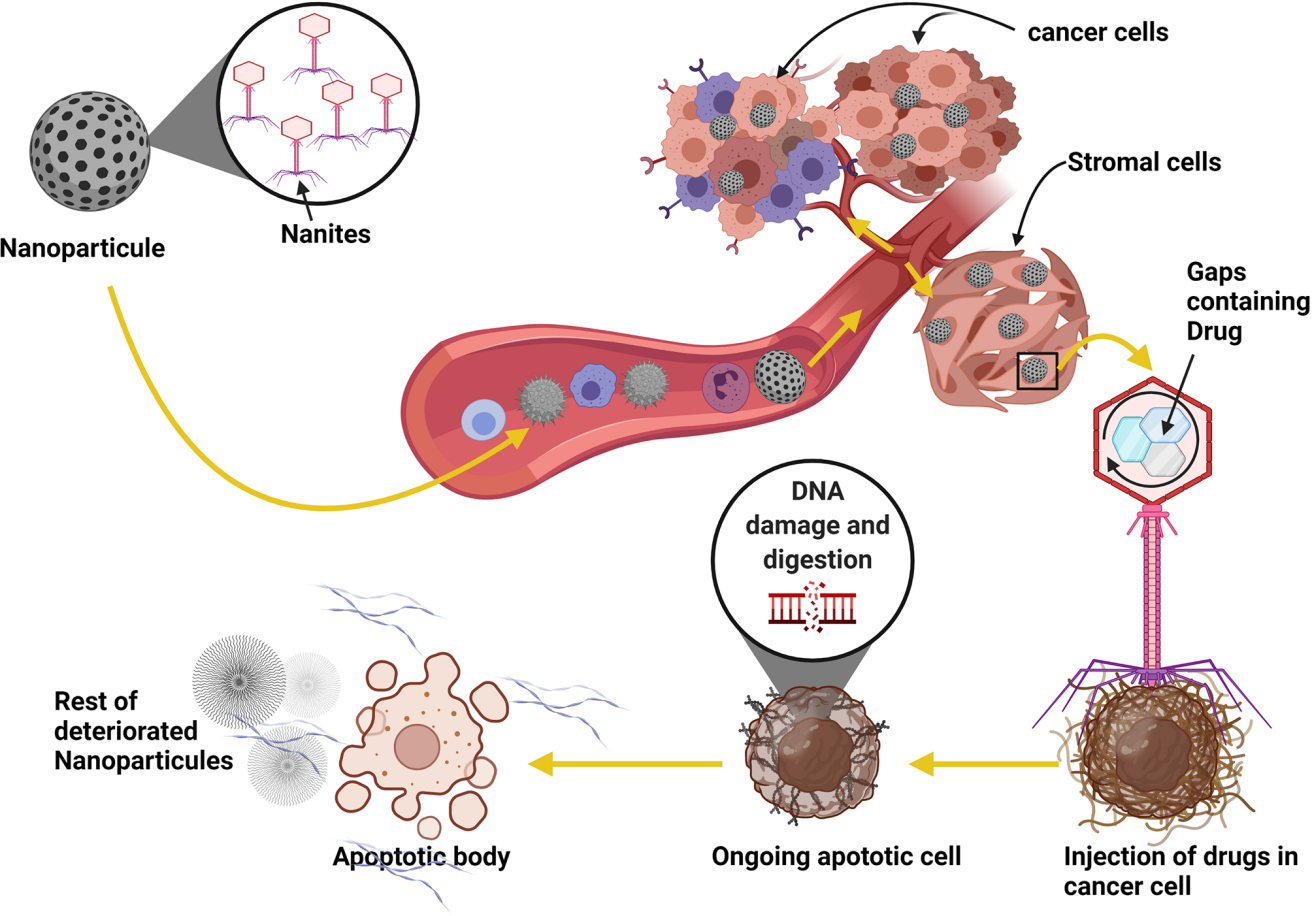
Using nanoparticles as a parasite in cancer therapy strategy. Patients with brain cancer can take the nanites as a pill. The nanites will enter into the blood circulation system using the digestif way. Once in the blood, nanites will target the cancer cells, parasitize them and inject their contents into the cells, resulting in the death of the host cells and the degradation of fibrin necessary for developing blood vessels and the development of cancer. The virus also uses the same process to target its host cell. The use of this method can significantly improve patient life and reduce cancer progression.

Recently, the use of *2-deoxy-D-glucose (2DG), 3-Bromopyruvate (3-BrOP)*, or *dichloroacetic acid (DCA)* have been shown to hinder the Warburg effect, thereby serving as blockades to metabolism alterations-induced immune cell reprogramming (165, 166). Also, nanoparticle delivery of *temozolomide (TMZ)* or other anti-cancer drugs with *DNA* fragmentation and digestion mechanisms have shown to be potent at inducing cancer cell death by reducing fibrin and collagen level in the cancer cell microenvironment to reduce angiogenesis and nutrient supplies (167, 168) (**Figure 5**).

The regulation of nutrient supplies to the tumor reduces their proliferation abilities *via* metabolism (glycolysis) control. Glioma stem cell metabolic heterogeneity (plasticity) accounts for heterogeneous tumors and cell populations. These metabolic and molecular heterogeneity results in cellular diversity, morphological modifications, and adaptations that enhance tumor recurrence after surgical resection, chemotherapy, and radiotherapy. With the knowledge of metabolic substrates preferences of cancer cells, therapeutic strategies can be targeted, impeding metabolites intake/assimilation or deploying *ATP* decoys such *ATPγS* or *ADPβS*, which are hydrolysis-resistant, thereby ultimately inhibiting tumor progression.

Additionally, the glioma cancer stem cell niche significantly contributes to their progression and resistance to therapies. Stroma constituted of nonmalignant cells (fibroblast, T-cells, macrophages, and endothelial cells) in the cancer microenvironment have been shown to affect the metabolic dynamics and tumor heterogeneity (169, 170). Notably, the existence of metabolic competition has been demonstrated between stroma and cancer cells (169, 171, 172). Besides, during migration and metastasis of cancerous cells, they require the association with stroma cells to adapt to the new environment (173–175). As such, it is suggested that pitching stroma against gliomas to compete for metabolites might be a therapeutic approach to inhibit the cancerous cells (176, 177). The use of the therapeutic approach of stromal cells against cancerous cells might reverse glioma phenotype and limit the symbiosis between cancer and stromal cell proliferation.

Furthermore, the fact that metabolic alteration induces reprogramming of immune cells (plasticity and polarization) as well (178, 179), suggests that hindering maladaptive modes of metabolism might utilize the synergy of slowing down tumor growth and enhancing immunosurveillance for detection and destruction of glioma cells. Reportedly, the immune cells (macrophages and T-cells) constituent in the stromal breaks the junctions between the tumors-associated cells and tumor cells and further cleanses the microenvironment, simultaneously avoiding inflammation (180, 181). Therefore, it might be necessary to utilize trained natural killer cells to target polymer between *cancer-associated fibroblasts (CAFs)* and macrophages and digest abnormal (tumor or tumor-associated) cells to discourage tumor growth. Emphatically, it is imperative to explore trained immune cells to target the strategic functions of glioma. Hence, exploring immunotherapy strategies coupled with other previously mentioned strategies might contribute significantly to the advancements in cancer therapeutics and patient survival.

## Conclusion

Immune cell plays an important role in carcinogenesis and hastening its progression. It is evident that the modification of cancer and immune cells’ plasticity contributes to tumor heterogeneity *via* the regulation of metabolism. Immune cells’ mobility in and out of blood vessels when they are activated scaffolds cancer cells metastasis and angiogenesis. Both immune and cancer cell reprogramming facilitated by *TGF alpha and beta* family members *via* hyperactivation of organelles (mitochondria, Golgi apparatus, and reticulum endoplasmic) and metabolism switches from anaerobic to aerobic glycolysis contribute to cancer progression, dampened immune response, and cancer resistance (182). Also, the alteration of cancer cells’ microenvironment constituents tends to create a niche that modifies the functions and induces plasticity of cells within this environment, thereby making the functions of the altered cells contribute to tumor development. The use of nanotechnology in brain cancer therapy has many advantages. The nanites can cross the brain cancer barrier without being repr ogrammed, as nanites do not undergo to metabolic process to contribute to tumor progression.

## Author Contributions

KouK drafted and wrote the manuscript. KonK constructed the graphical illustrations. GA and AA proofread the manuscript. PK, XG, XY and DG read and approved the manuscript for submission. All authors contributed to the article and approved the submitted version.

## Funding

Research in our laboratories is funded by the National Natural Science Foundation of China (Grant numbers: 81772688 and 81372698). China and Jiangsu Postgraduate Science Foundation-funded project (2013 M540466 and 1301068C), Qing Lan Project, and a project Priority Academic Program Development of Jiangsu Higher Education Institutions (PAPD).

## Conflict of Interest

The authors declare that the research was conducted in the absence of any commercial or financial relationships that could be construed as a potential conflict of interest.

## Publisher’s Note

All claims expressed in this article are solely those of the authors and do not necessarily represent those of their affiliated organizations, or those of the publisher, the editors and the reviewers. Any product that may be evaluated in this article, or claim that may be made by its manufacturer, is not guaranteed or endorsed by the publisher.

## Supplementary Material

Supplementary Figure 1
**(A)** Immune and cancer interaction in brain cancer development and metastasis. In the presence of an abnormal cell or pathogen, the immune cells react to stop the proliferation and spread of the cancer cell or pathogen. The cancer cell develops on their membrane certain receptors that protect them against the immune response. The immune cell did not see cancer cells as a potential danger; instead of destroying the isolated cancer cell, the immune cell aids its tissue colonization to increase its chance to survive. This insertion leads to inflammation of the tissue and the synthesis of cytokines. The synthesis of cytokines leads to cancer cell proliferation and angiogenesis and enhances cancer cell metastasis via intussusception of blood vessels. The cancer cell colony pressure causes intussusception of blood vessels. Weaken blood vessels become more permeable to the immune and cancer cells, thereby facilitating cancer cell metastasis inside the brain. **(B)** Mechanism used by the cancer cell to escape immune responses. Each cell has many receptors on its membrane important for signal induction and recognition between cells and immune cells. In the normal and immune cell interaction, the immune cell recognizes the normal cell via direct binding of the receptor **(A–E)**. In the interaction with cancer cells, there is an intermediate protein (TGFβ, GDNF, and Calcium), the binding of intermediate protein on cancer cell receptors induces a cascade of reactions inside the cancer cell. The binding of immune cells to cancer cells through the receptor via TGF protein (red) activates the metabolic signaling pathway in both cancer and immune cells leading to tumor progression.

Supplementary Figure 2 Immune cell lineage and cancer cell reprogramming through the Warburg effect and oncogene upregulation. All immune cell lineage contributes to cancer development. When the immune cells enter the cancer microenvironment, their metabolism changes, and their functions (plasticity and polarization) are altered. The metabolic modification contributes to the infiltration of cancer and immune cell to tumor tissue via the upregulation of cytokines such as interleukins (IL-1α, IL-6, IL-8, IL-10), oncogene such as (TGFα/β, VEGF), and tumor necrosis factor (TNF-α). The combined upregulation of oncogenes and metabolism/Warburg effect promotes cancer cell proliferation, metastasis, and malignancy.

Supplementary Figure 3 epigenetic alteration and oncogene overexpression due to metabolic changes and activation of PI3K/AKT/mTOR pathways. Metabolism is one of the principal cell dynamics that ensure energy for cell division and survival. During inflammation, the overexpression of certain growth factors activates the PI3K/AKT/mTOR pathway, which controls several genes to perform their tasks. The binding of TGF-β family members on their receptors activates PI3K and aerobic glycolysis in cancer and immune cells. In cancer cells, the binding of TGF-β on their receptor activates PI3K/AKT pathways that simultaneously control the activities of mTOR and GSK3β genes. The activation of AKT modulates the activity of GSK3β and activates the mTOR pathway to regulate mitochondrial glycolysis. When glycolysis is insufficient for glioma cell proliferation and survival, this pathway can be switched using calcium (Ca2+) ions to change from one pathway to another, making the PI3K/AKT pathway complicated. In immune cells, during inflammation, the activation of macrophages also activates the PI3K/AKT/mTOR pathway activating HIF-1β, which causes glycolysis and increases mitochondrial activity. In both cases, the activation of glycolysis significantly affects oncogenes gene expression by encouraging epigenetic modifications such as histones acetylation and DNA methylation (H3K4 for SOX1 and H3K9 for GDNF) to promote glioma.

## Abbreviations

GDNF, Glial cell line-derived neurotrophic factor; SOX1, Sex-determining region Y box1; TGF-beta, Transforming Growth Factor beta; EGFR, epidermal growth factor receptor; PI3-K, phosphatidylinositide 3-kinase; STAT3, signal transduction activator of transcription 3; GSK3 beta, Glycogen synthase kinase 3 beta; PTEN, Phosphatase and Tensin, VEGF, Vascular endothelial growth factor; TMZ, temozolomide; CAF, Cancer-Associated Fibroblast; IL-6, interleukins and TNF-α, tumor necrosis factor-alpha.
