# Supplementary figures and images for: Cancer Metabolism: The Role of Immune Cells Epigenetic Alteration in Tumorigenesis, Progression, and Metastasis of Glioma
Source: Front Immunol. 2022 Mar 22;13:831636. doi: 10.3389/fimmu.2022.831636 (PMC8980436; doi:10.3389/fimmu.2022.831636)

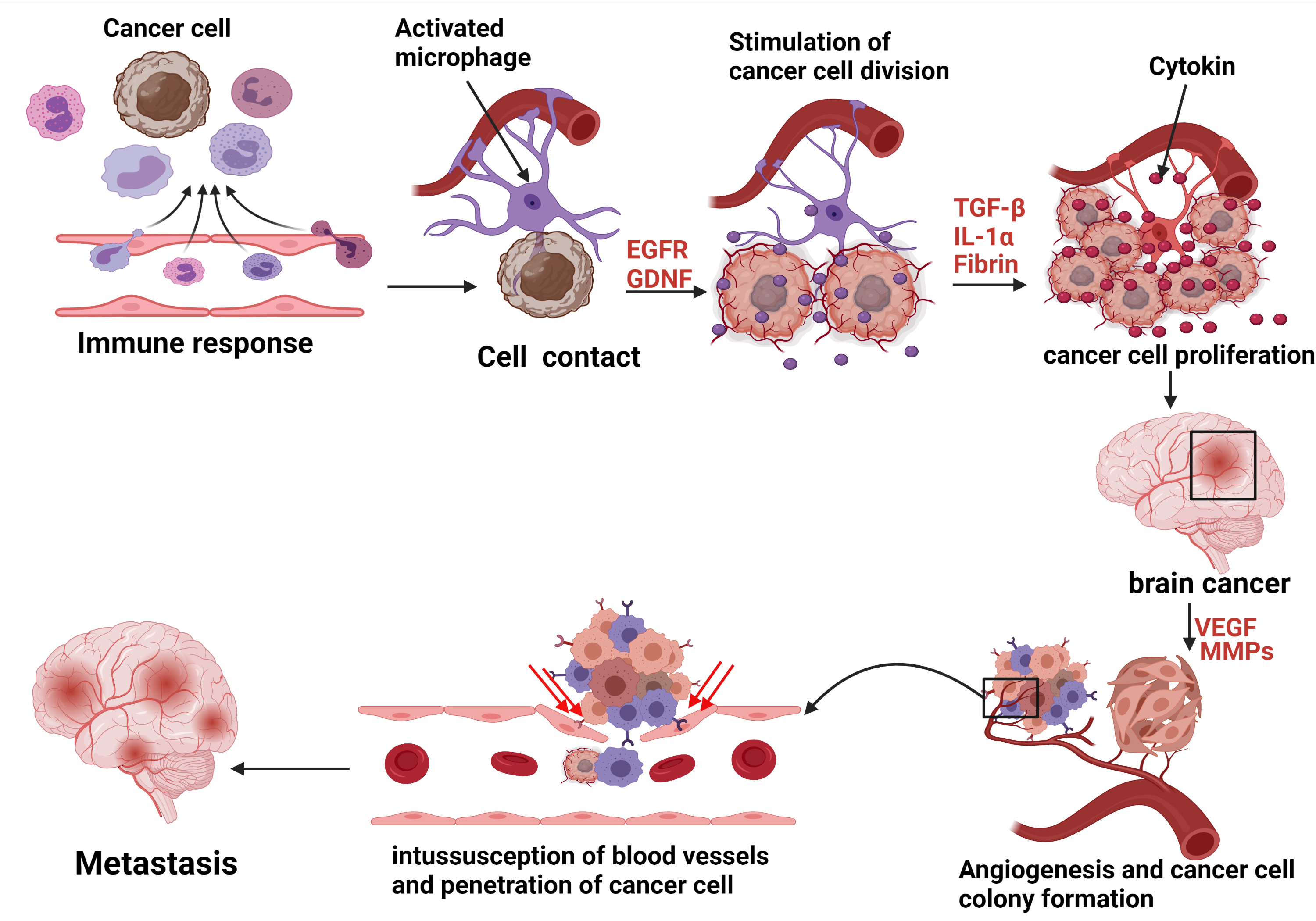

Supplement: Supplementary Figure 1 — (A) Immune and cancer interaction in brain cancer development and metastasis. In the presence of an abnormal cell or pathogen, the immune cells react to stop the proliferation and spread of the cancer cell or pathogen. The cancer cell develops on their membrane certain receptors that protect them against the immune response. The immune cell did not see cancer cells as a potential danger; instead of destroying the isolated cancer cell, the immune cell aids its tissue colonization to increase its chance to survive. This insertion leads to inflammation of the tissue and the synthesis of cytokines. The synthesis of cytokines leads to cancer cell proliferation and angiogenesis and enhances cancer cell metastasis via intussusception of blood vessels. The cancer cell colony pressure causes intussusception of blood vessels. Weaken blood vessels become more permeable to the immune and cancer cells, thereby facilitating cancer cell metastasis inside the brain. (B) Mechanism used by the cancer cell to escape immune responses. Each cell has many receptors on its membrane important for signal induction and recognition between cells and immune cells. In the normal and immune cell interaction, the immune cell recognizes the normal cell via direct binding of the receptor (A–E). In the interaction with cancer cells, there is an intermediate protein (TGFβ, GDNF, and Calcium), the binding of intermediate protein on cancer cell receptors induces a cascade of reactions inside the cancer cell. The binding of immune cells to cancer cells through the receptor via TGF protein (red) activates the metabolic signaling pathway in both cancer and immune cells leading to tumor progression. [file Image_1.jpeg]

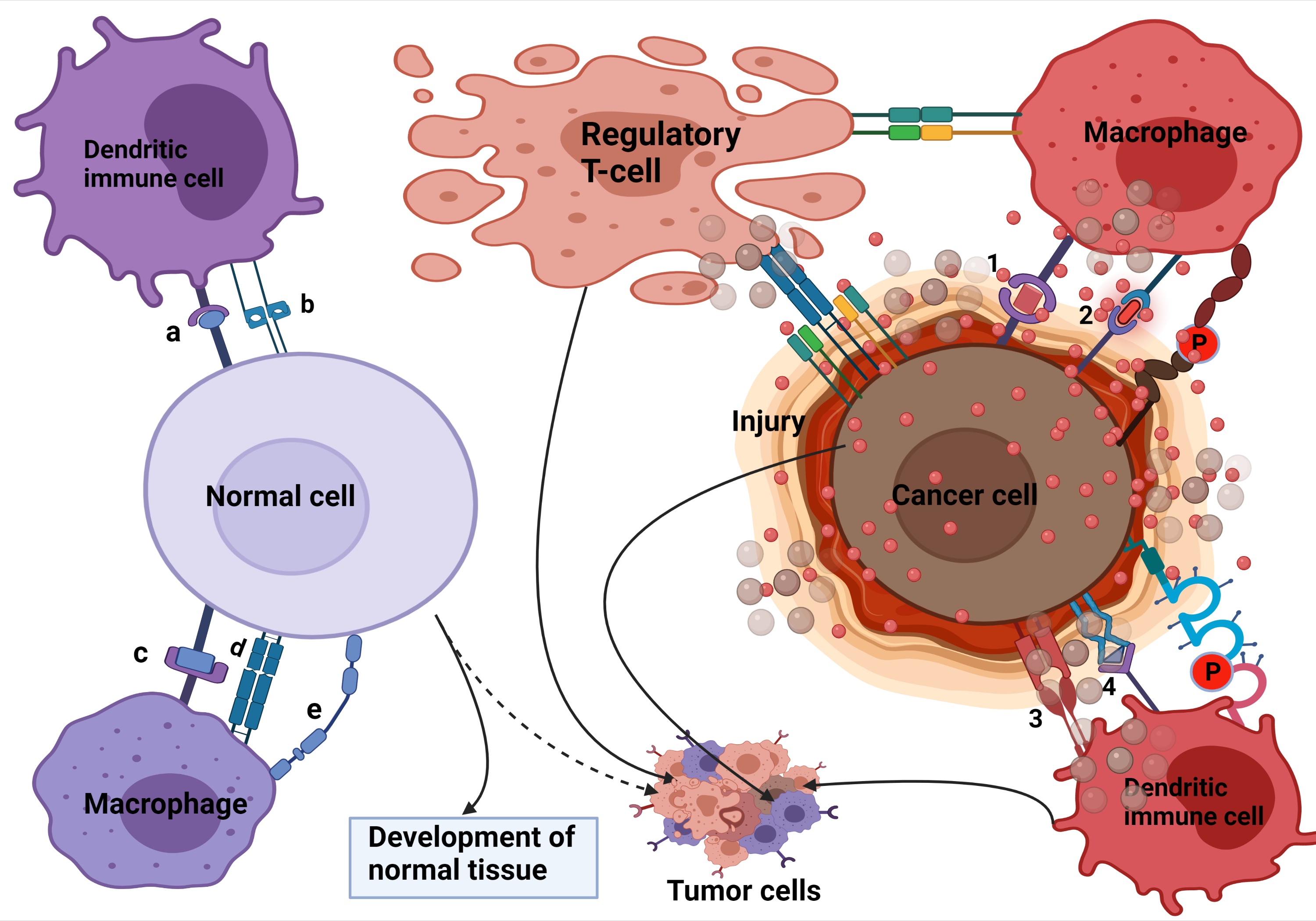

Supplement: Supplementary Figure 2 — Immune cell lineage and cancer cell reprogramming through the Warburg effect and oncogene upregulation. All immune cell lineage contributes to cancer development. When the immune cells enter the cancer microenvironment, their metabolism changes, and their functions (plasticity and polarization) are altered. The metabolic modification contributes to the infiltration of cancer and immune cell to tumor tissue via the upregulation of cytokines such as interleukins (IL-1α, IL-6, IL-8, IL-10), oncogene such as (TGFα/β, VEGF), and tumor necrosis factor (TNF-α). The combined upregulation of oncogenes and metabolism/Warburg effect promotes cancer cell proliferation, metastasis, and malignancy. [file Image_2.jpeg]

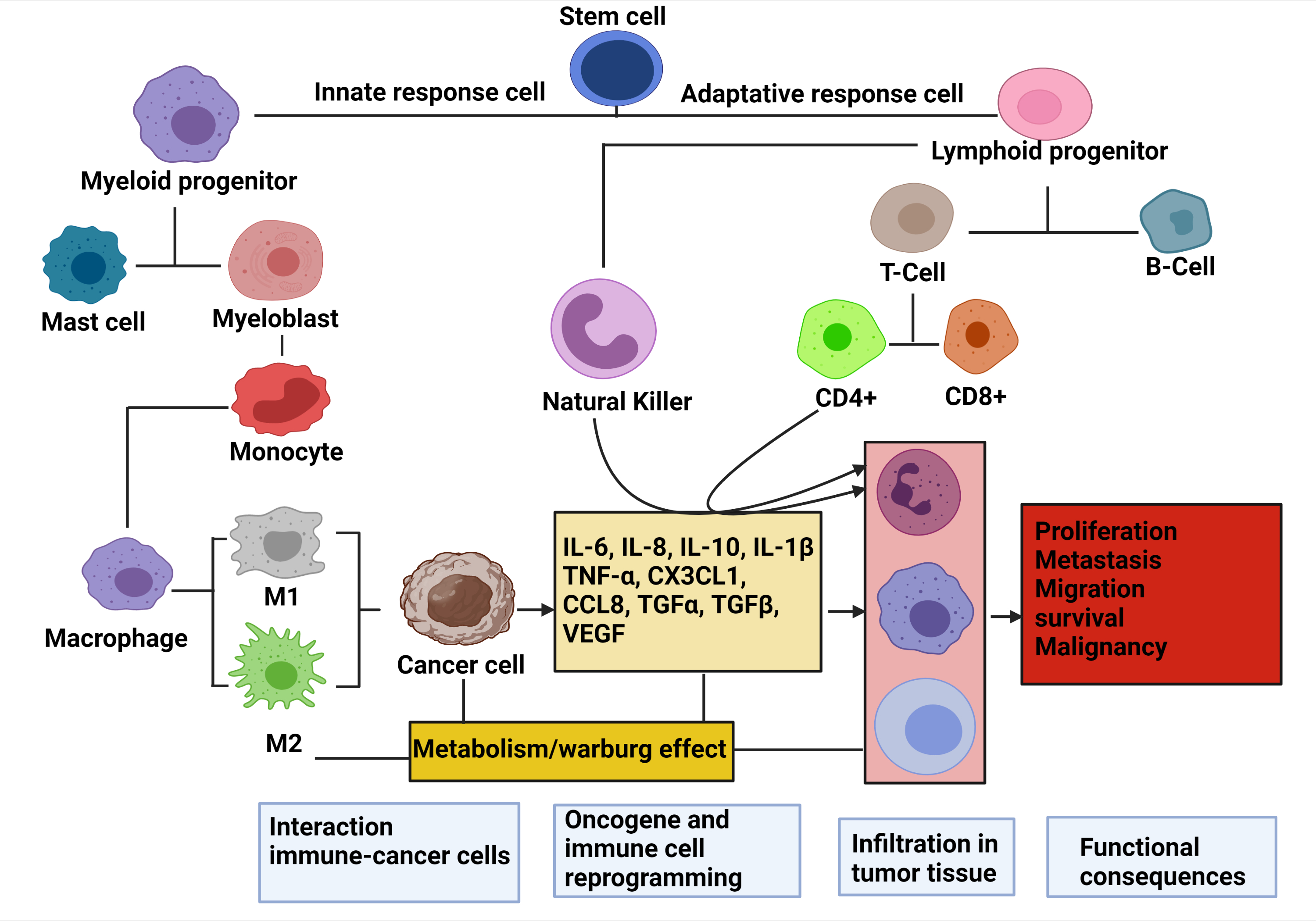

Supplement: Supplementary Figure 3 — epigenetic alteration and oncogene overexpression due to metabolic changes and activation of PI3K/AKT/mTOR pathways. Metabolism is one of the principal cell dynamics that ensure energy for cell division and survival. During inflammation, the overexpression of certain growth factors activates the PI3K/AKT/mTOR pathway, which controls several genes to perform their tasks. The binding of TGF-β family members on their receptors activates PI3K and aerobic glycolysis in cancer and immune cells. In cancer cells, the binding of TGF-β on their receptor activates PI3K/AKT pathways that simultaneously control the activities of mTOR and GSK3β genes. The activation of AKT modulates the activity of GSK3β and activates the mTOR pathway to regulate mitochondrial glycolysis. When glycolysis is insufficient for glioma cell proliferation and survival, this pathway can be switched using calcium (Ca2+) ions to change from one pathway to another, making the PI3K/AKT pathway complicated. In immune cells, during inflammation, the activation of macrophages also activates the PI3K/AKT/mTOR pathway activating HIF-1β, which causes glycolysis and increases mitochondrial activity. In both cases, the activation of glycolysis significantly affects oncogenes gene expression by encouraging epigenetic modifications such as histones acetylation and DNA methylation (H3K4 for SOX1 and H3K9 for GDNF) to promote glioma. [file Image_3.jpeg]

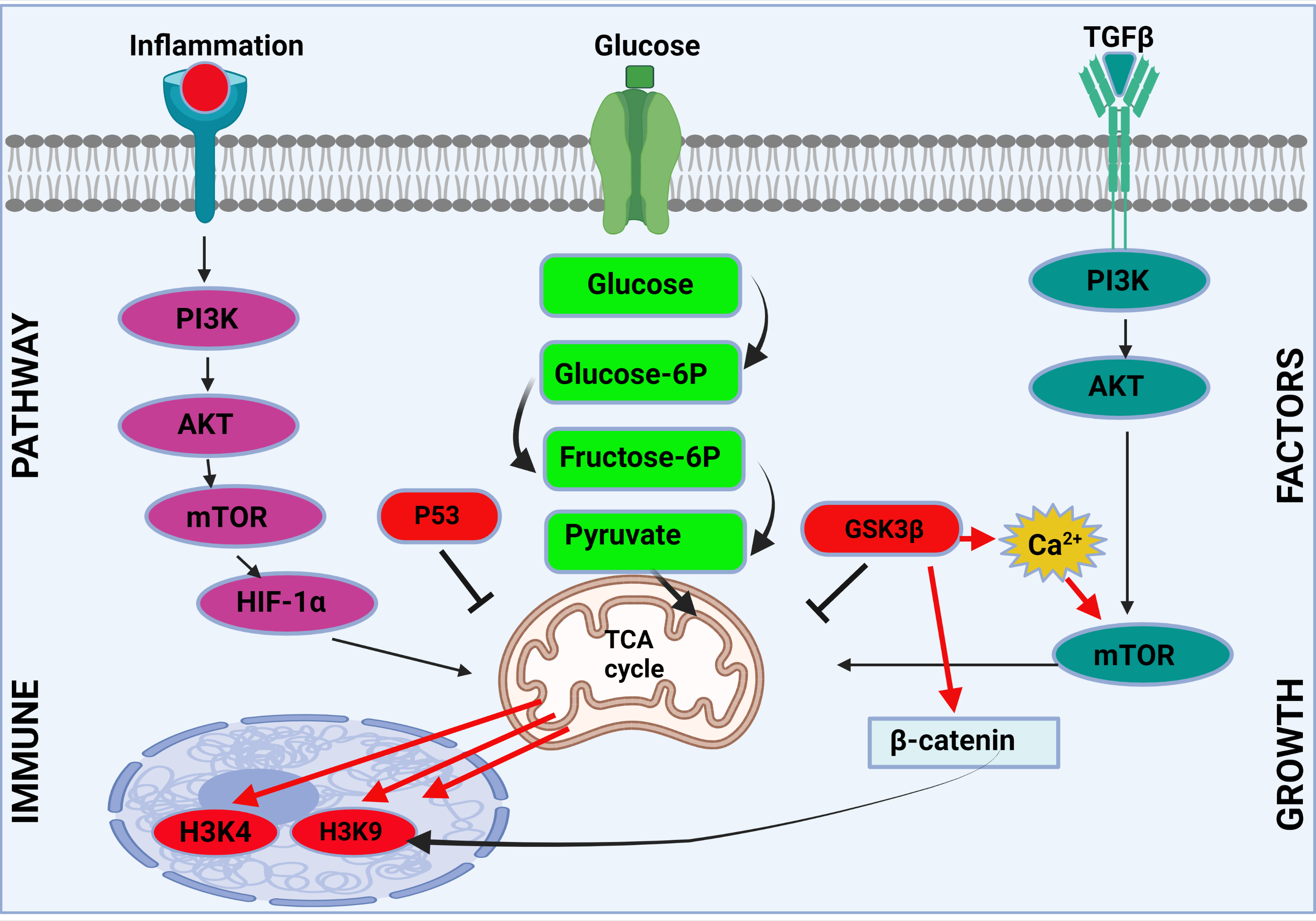

Supplement: Supplementary file 4 [file Image_4.jpeg]
